# BAL fluid cells in newly diagnosed pulmonary sarcoidosis with different clinical activity

**DOI:** 10.1080/03009730802579729

**Published:** 2009-02-04

**Authors:** Edvardas Danila, Laimutė Jurgauskienė, Jolita Norkūnienė, Radvilė Malickaitė

**Affiliations:** ^1^Clinic of Chest Diseases, Allergology and Radiology, Vilnius UniversityVilniusLithuania; ^2^Centre of Pulmonology and Allergology, Vilnius University Hospital Santariškių klinikosVilniusLithuania; ^3^Clinic of Cardiovascular Diseases, Vilnius UniversityVilniusLithuania; ^4^Laboratory of Clinical Immunology at the Centre of Laboratory Diagnostics, Vilnius University Hospital Santariškių klinikosVilniusLithuania; ^5^Department of Mathematical Statistics, Vilnius Gediminas Technical UniversityVilniusLithuania; ^6^Vilnius College of Higher EducationVilniusLithuania

**Keywords:** Bronchoalveolar lavage, bronchoscopy, sarcoidosis

## Abstract

**Background:**

Sarcoidosis is associated with an increase in the number of alveolar T cells (CD3^+^ cells) and an increase of the CD3^+^CD4^+^ lymphocyte subset. However, the number of lymphocytes and the CD4/CD8 ratio in bronchoalveolar lavage (BAL) fluid are highly variable in sarcoidosis. Comparative studies have demonstrated that geographic and ethnic factors are linked to the specific characteristics of patients with sarcoidosis.

**Aim of the study:**

To investigate peculiarities of BAL fluid (BALF) cell patterns in different clinical activity of pulmonary sarcoidosis at the time of diagnosis.

**Material and methods:**

A total of 308 non-treated patients (138 asymptomatic and 170 with sarcoidosis-related symptoms) and 40 previously empirically steroid-treated patients with newly diagnosed sarcoidosis have been prospectively examined.

**Results:**

Significant BAL fluid lymphocytosis and increased CD4/CD8 ratio were characteristic for all three sarcoidosis patient groups. A total of 12% of asymptomatic patients, 3% of patients with sarcoidosis-related symptoms, and 5% of previously treated symptomatic patients had normal BALF cell counts. Non-treated patients with sarcoidosis-related symptoms had significantly higher lymphocytosis (45±19% versus 39±17%, *P*<0.01), CD4/CD8 ratio (9.3±5.0 versus 5.7±4.5, *P*<0.001), and total BALF cell count (411±322 106/mL versus 334±273 106/mL, *P*<0.05), compared with asymptomatic patients. However, previously treated symptomatic patients had lower lymphocytosis (39±15% versus 45±19%, *P*=0.058), and total BALF cell count (292±166 106/mL versus 411±322 106/mL, *P*<0.05) compared with non-treated symptomatic patients. The same trend was noticed for CD4/CD8 ratio (8.3±4.8), although a statistically significant difference was not achieved.

**Conclusions:**

Independently of clinical symptoms at the time of diagnosis sarcoid patients have significantly different BAL fluid cell patterns compared to healthy persons. BAL fluid cell changes are more prominent in corticosteroid non-treated patients with clinically active sarcoidosis. Treatment with systemic corticosteroids may modify typical BALF cellular patterns of sarcoidosis.

## Introduction

Sarcoidosis is a systemic granulomatous disease of unknown aetiology that primarily affects the lungs, although multiorgan involvement frequently occurs. It is likely that the expression of the disorder in response to inciting agent or agents is modified by the genetics of the host immune response. Although many clinical features and associated syndromes have been described, the phenotype of sarcoidosis at the time of diagnosing provides only limited information about the aetiology or pathogenesis of the disorder (1). Sarcoidosis is the most frequently observed interstitial lung disease of unknown origin. The clinical manifestations are largely non-specific, as are the histological features (2,3). The clinical features of sarcoidosis are varied, but at either end of the range they are an acute form (Löfgren's syndrome) and chronic sarcoidosis (4).

Sarcoidosis is associated with an increase in the number of alveolar T cells, and a shift to an increase in CD4 cells within these cells can be observed (5). Bronchoalveolar lavage (BAL) is a method of sampling fluid and cells from a large area of the lung tissue by instilling and aspirating saline via a bronchoscope wedged in bronchi (4). The increase in BAL lymphocytes and the CD4/CD8 ratio of BAL lymphocytes is a typical feature of sarcoidosis (6). However, the number of lymphocytes as well as the CD4/CD8 ratio in BAL fluid (BALF) are highly variable in sarcoidosis (7).

Comparative epidemiological studies have demonstrated that geographic, ethnic, and genetic factors are linked to the specific clinical characteristics of sarcoid patients (8–10).

The present study aimed to investigate peculiarities of BALF cell patterns in different clinical activity of pulmonary sarcoidosis at the time of diagnosis in a large group of sarcoid patients (*n*=348). Only a few studies (11,12) were devoted to this issue. In some of them (13) the number of subjects was small. This is the first study designed to evaluate this problem among the Lithuanian population. Additionally, in this study the data from BALF cell examinations in corticosteroid-treated and non-treated patients with sarcoidosis were compared. To our knowledge such a study has not been published earlier.

## Material and methods

This is a prospective study of patients with a newly diagnosed sarcoidosis. It is a part of the first large sarcoidosis study in Lithuania launched in 1993. The patients underwent diagnostic tests only as part of routine clinical investigation. The study population consisted of 348 sarcoid patients. The patients were separated into three groups. The first group consisted of 138 asymptomatic non-treated patients; the second group consisted of 170 non-treated patients who had sarcoidosis-related, mostly Löfgren's syndrome symptoms; and the third group (a subgroup of the symptomatic patients) consisted of 40 patients who had been previously empirically steroid-treated in other institutions (1–3 weeks, 20–30 mg daily of prednisone or its equivalent) mainly because of erythema nodosum and joint pains. All of them visited the Centre of Pulmonology and Allergology of Vilnius University Hospital *Santariškių klinikos* in the period of 1995 to 2007. Sarcoidosis was diagnosed according to World Association of Sarcoidosis and Other Granulomatous Diseases (WASOG) guide-lines (14).

None of the patients had any relevant medical history or comorbidity (e.g. tuberculosis). No patient had a history of exposure to organic or mineral dust that is known to cause granulomatous lung disease. The demographic data of the study population are summarized in Table I.

**Table I.** Demographic data of the study patient population.

Demographics	Asymptomatic	Symptomatic	Treated
Subjects, *n*	138	170	40
Age, years	35±9	38±11	39±10
Male/female, *n*	79/59	45/125	12/28
Non-smokers/smokers	106/32	145/25	36/4
FVC,% predicated	101±15	105±16	105±16
FEV_1_,% predicated	99±17	100±15	100±16
FEV_1_/FVC	81±9	81±7	82±8
VC,% predicated	102±16	106±16	108±13
TLC,% predicated	97±11	96±14	100±12
DL_CO_,% predicated	88±12	86±15	85±16

Data are presented as mean±standard deviation, and numbers (*n*). FVC = forced vital capacity; FEV_1_=forced expiratory volume in one second; VC = vital capacity; TLC = total lung capacity; DL_CO_=diffusing capacity of carbon monoxide.

The control group consisted of 55 healthy volunteers (35 non-smokers and 20 smokers). A signed informed consent form was obtained from all participants. The study has been approved by the Committee on Biomedical Ethics of the Vilnius University Hospital *Santariškių klinikos*.

Fibre-optic bronchoscopy and BAL were performed as described elsewhere (15). The subjects were premedicated with atropine, and lidocaine was delivered topically via an atomizer. The bronchoscope was inserted transnasally (in most cases) or orally and passed to segmental or subsegmental bronchi. BAL was performed in the right middle lobe, lingual, or in the area of greatest radiological abnormality. Sterile isotonic saline at room temperature was instilled in two 50 mL aliquots. Each aliquot was retrieved with gentle manual aspiration. Only the second aliquot was analysed. We have used the same BAL as well as BALF analysis method all through the period of the study.

BALF cells were examined at the Laboratory of Clinical Immunology at the Centre of Laboratory Diagnostics of Vilnius University Hospital *Santariškių klinikos.* BALF for cell analysis was filtered through a 70 mm pore filter to remove mucus, and then cellular material was sedimented by centrifugation (300 *g* for 10 min at 4°C). Differential cell counts of 600 BALF cells were performed on May-Grunwald-Giemsa or similar stained preparations. Viability of BALF cells was determined by 0.4% trypan blue dye exclusion (this method is based on the principle that live cells do not take up certain dyes, whereas dead cells do). For lymphocyte subpopulation analysis 100 µL of cell suspensions (1×10^6^ cells) were incubated for 15 min at 4°C with 20 µL fluorescein isothiocyanate (FITC) or phycoerythrin (PE) conjugated monoclonal antibody (Becton Dickinson). Following the incubation, 2 mL of cold phosphate buffered saline (PBS) was added to the pellet and washed at 300 *g* for 10 min at 4°C. Prepared BALF samples were examined by flow cytometry (FACS Calibur, Becton Dickinson).

### Statistical methods

Statistical data were processed at the Department of Mathematical Statistics of Vilnius Gediminas Technical University. Statistical data processing was performed by SPSS 15.0 Program. The data of different groups were compared according to Student's *t* test and Mann-Whitney U-test. Correlation was evaluated by the Pearson correlation coefficient. In all tests a *P*-value of <0.05 was considered to be statistically significant.

## Results

**Figure 1.**
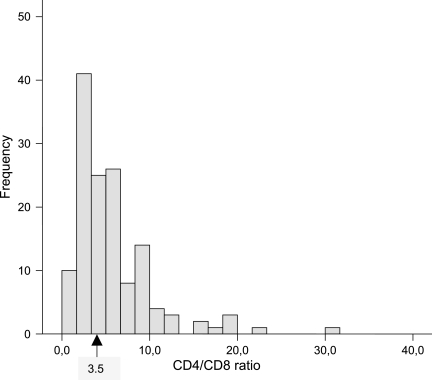
Distribution of CD4/CD8 ratio in bronchoalveolar lavage fluid (BALF) of asymptomatic sarcoid patients.

**Figure 2.**
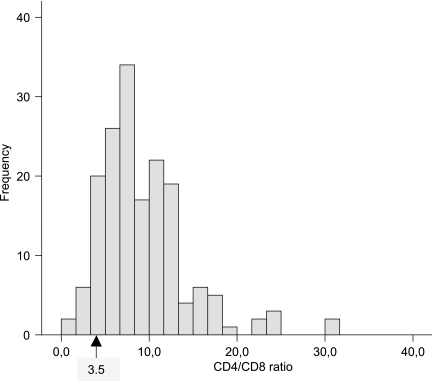
Distribution of CD4/CD8 ratio in bronchoalveolar lavage fluid (BALF) of sarcoid patients with clinical symptoms.

**Figure 3.**
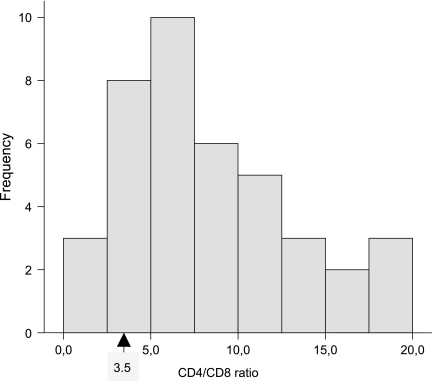
Distribution of CD4/CD8 ratio in bronchoalveolar lavage fluid (BALF) of previously treated sarcoid patients with clinical symptoms.

The volume of recovered BAL fluid was 65±10 mL. The percentage of the viability of the bronchoalveolar lavage fluid cells was 97±3%. Significant BAL fluid lymphocytosis and increased CD4/CD8 ratio were characteristic for all three sarcoidosis patient groups (Table II). Normal (near-normal) BAL fluid cell percentages were found in a minority of the patients. Normal BALF cell counts were found in 12% of asymptomatic patients (Figure 1), 3% of patients with sarcoidosis-related symptoms (Figure 2), and 5% of previously treated symptomatic patients (Figure 3).

**Table II.** Characteristics of bronchoalveolar lavage fluid (BALF) cell counts of patients and healthy volunteers.

Cells	Healthy (*n*=55)	Asymptomatic (*n*=138)	Symptomatic (*n*=170)	Treated (*n*=40)
Total cells (×10^6^/mL)	254±247	334±273^f^	411±322^b^	292±166^f^
Macrophages, %	79±8^a^	56.4±17^e^	49.3±20	55.5±15
Lymphocytes, %	15.7±7^a^	39±17^e^	45±19	39±15
Neutrophils, %	5±2	4±4	5±5	5±3
Eosinophils, %	0.3±04	0.6±1^c^	0.7±0.9^a^	0.5±0.7^c^
CD4, %	44±13^a^	72±15^d^	82±13	80±12
CD8, %	32±13^a^	17±8^d^	12±7	13±7
CD4/CD8	1.7±1.0^a^	5.7±4.5^d^	9.3±5.0	8.3±4.8

Data are presented as mean±standard deviation. The comparison was done for the three patient groups separately. ^a^ *P*<0.001 healthy volunteers versus patients. ^b^ *P*<0.01 healthy volunteers versus patients. ^c^ *P*<0.05 healthy volunteers versus patients. ^d^ *P*<0.001 symptomatic non-treated patients versus other patients. ^e^ *P*<0.01 symptomatic non-treated patients versus other patients. ^f^ *P*<0.05 symptomatic non-treated patients versus other patients.

Macrophage-lymphocyte rosettes (adherence of lymphocytes to alveolar macrophages) and Langhans-type multinucleated giant cells were observed in BALF of all patient groups, but not in healthy volunteers’ BALF samples. Macrophage-lymphocyte rosettes were found in 64%, 72%, and 75% of asymptomatic patients, patients with symptoms, and treated patients, respectively, and Langhans-type giant cells in 7%, 15%, and 10% of cases respectively.

Non-treated patients with sarcoidosis-related symptoms had significantly higher lymphocytosis and CD4/CD8 ratios as well as total BALF cell count and giant cells in BALF, compared with asymptomatic patients. However, previously treated symptomatic patients had lower lymphocytosis (*P*=0.058) and total BALF cell count, compared with non-treated symptomatic patients. The same trend was seen for CD4/CD8 ratios, although statistically significant difference was not achieved.

Evaluating sarcoid patients, a weak negative correlation was observed comparing the presence of symptoms of acute sarcoidosis (Löfgren's syndrome) with BALF neutrophils (*r*= − 0.26, *P*<0.001) and eosinophils (*r*= − 0.27, *P*<0.001). Furthermore, BAL fluid neutrophils and eosinophils correlated negatively—weakly but statistically significantly—with pulmonary function indices. Neutrophils correlated with forced vital capacity (FVC) and vitalcapacity (VC) (both *r*= − 0.26, *P*<0.05) and forced expiratory volume in one second (FEV_1_)/FVC (*r*= − 0.24, *P*<0.05), and eosinophils with FVC (*r*= − 0.26, *P*<0.05), FEV_1_ (*r*= − 0.3, *P*<0.01), and VC (*r*= − 0.3, *P*<0.05) in asymptomatic patients. Neutrophils correlated with FVC (*r*= − 0.32, *P*<0.001), VC (*r*= − 0.27, *P*<0.01), and FEV_1_ (*r*= − 0.33, *P*<0.001), and eosinophils with FVC (*r*= − 0.26, *P*<0.01), VC (*r*= − 0.28, *P*<0.01), and total lung capacity (TLC) (*r*= − 0.32, *P*<0.01) in symptomatic patients.

## Discussion

This study evaluated the bronchoalveolar lavage fluid cells in different clinical activity of pulmonary sarcoidosis at the time of diagnosis. Data of examination of BALF cells of corticosteroid − treated and non-treated patients with sarcoidosis were compared.

The major findings in our study are: 1) independently of presence of clinical symptoms, a significantly increased number of BAL fluid lymphocytes and an increased ratio of CD4/CD8 T cells compared with healthy subjects were found; 2) patients with sarcoidosis-related clinical symptoms had significantly higher BAL fluid lymphocytes count and CD4/CD8 ratio compared with asymptomatic patients; 3) the number of BALF total cells and lymphocytes of patients previously treated with systemic corticosteroids was lower compared with non-treated patients.

Key findings of our study—an increased number of BALF lymphocytes and CD4/CD8 ratio among the Lithuanian population—are in line with previous publications (12,16). Changes of BALF cells reflect populations of immune cells accumulated in the lung parenchyma. Sarcoidosis is a granulomatous disorder resulting from an uncontrolled cell-mediated immune reaction characterized by accumulation of activated monocytes/macrophages and CD4 T lymphocytes in all sites of disease activity. In the lung, this accumulation in both air spaces and interstitium (alveolitis) precedes and accompanies the development of granulomas (17). The majority of our patients had macrophage-lymphocyte rosettes and Langhans-type giant cells in the BAL fluid. These cells reflected lung parenchyma granulomas (18), structured masses composed of macrophage-derived cells (which assume an epithelioid aspect) and of lymphocytes (19).

We have found that the clinical symptoms of our study patients are associated with changes of BAL fluid cell populations. Similar to our findings, Valeyre et al. (13) found a large increase in the percentage of lymphocytes in BAL fluid of sarcoid patients with Löfgren's syndrome, and Drent et al. (12) identified that patients with erythema nodosum and/or arthralgia and hilar lymphadenopathy have the highest CD4/CD8 ratio compared with other sarcoid patients. Ward et al. (11) found that the lavage T lymphocytes percentage and CD4/CD8 ratio were highest in patients with sarcoidosis studied soon after an acute onset with an inflammatory condition such as erythema nodosum or uveitis.

A variability of BALF cell populations in every group of patients was found. As shown in Figure 1 and Figure 2, the variability was higher in the group of asymptomatic patients. A CD4/CD8 ratio >3.5, which is a breaking-point for sarcoidosis differential diagnostics (20), was found in a much greater part of patients with sarcoidosis-related symptoms compared to asymptomatic patients. Furthermore, significantly more asymptomatic patients had normal BALF cell percentages. The explanation of this phenomenon may be different at the time of the beginning of the disease. The time point of the onset of the disease for the individual patient, with the exception of patients with Löfgren's syndrome, is generally not known. It is likely that the duration of the disorder for a substantial part of asymptomatic patients in our study group was at least several months. However, it seems that the disorder had not continued for one year, as the number of neutrophils and/or eosinophils in BALF was not high. Other authors (21,22) have indicated that an elevated count of these cells in BAL fluid is associated with more advanced and severe sarcoidosis, although markers for disease progression to fibrosis are lacking (23). We have found previously a significant decrease of BAL fluid CD4/CD8 ratio with increasing radiographic stage of sarcoidosis (24).

Macrophage-lymphocyte rosettes and giant cells (elements of immune granuloma) were found more often in BALF of both symptomatic patient groups compared to asymptomatic patients. This finding may reflect still on-going inflammation in the lung parenchyma. Macrophages and T lymphocytes from sarcoid patients are activated, and they release several mediators which recruit other immune cells to the lung parenchyma (19). It is suggested that apoptosis (programmed cell death) may be associated with the course of granulomatous inflammation in pulmonary sarcoidosis (25). These processes depend on various, mostly unknown, genetic and environmental factors simultaneously (26,27). The influence of ethnic and genetic factors determines the heterogeneous clinical course of sarcoidosis (17).

Milder signs of active alveolitis (e.g. total cell count, number of lymphocytes) in BAL fluid of treated patients compared to non-treated patients were found. Corticosteroids suppress the granuloma formation and reverse consecutive clinical, radiographical, functional and biological manifestations. However, corticosteroid therapy is not curative and seems not effective for the natural course of the disease (17). We do not agree with the still occurring practice of empirical treatment with corticosteroids of patients with possible sarcoidosis. As our data show, BAL fluid cell patterns may become non-typical for sarcoidosis in such cases, and further diagnostics may be complicated.

## Conclusions

1. Independently of clinical symptoms at the time of diagnosis sarcoid patients have significantly different BAL fluid cell patterns compared to healthy persons.
2. BAL fluid cell changes are more prominent for corticosteroid non-treated patients with clinically active sarcoidosis.
3. Treatment with systemic corticosteroids may modify typical BALF cellular patterns of sarcoidosis.
